# The surveillance of vero cytotoxin-producing Escherichia coli O157 in Wales, 1990 to 1998.

**DOI:** 10.3201/eid0504.990422

**Published:** 1999

**Authors:** R M Chalmers, S M Parry, R L Salmon, R M Smith, G A Willshaw, T Cheasty

**Affiliations:** Public Health Laboratory Service Communicable Disease Surveillance Centre, Cardiff, United Kingdom. rachel.chalmers@cdsc.wales.nhs.uk

## Abstract

Population-based surveillance for Vero cytotoxin-producing Escherichia coli (VTEC) O157 has been carried out in Wales since 1990. The annual incidence has remained stable during the 9-year period (mean: 1.6 cases per 100,000 population); the rate is highest in children younger than 5 years of age. Blood in the stool is reported in fewer than half the cases, indicating the importance of screening all fecal specimens for VTEC O157.


### The Surveillance of Vero Cytotoxin-Producing *Escherichia coli* O157 in Wales, 1990 to 1998

**Rachel M. Chalmers,* Sharon M. Parry,† Roland L. Salmon,* Robert M.M. Smith,* Geraldine A. Willshaw,‡ and Tom Cheasty‡**
*Public Health Laboratory Service Communicable Disease Surveillance Centre, Cardiff, United Kingdom; †Welsh Combined Centres for Public Health, Cardiff, United Kingdom; ‡Central Public Health Laboratory, Colindale, London, United Kingdom

Population-based surveillance for Vero cytotoxin-producing *Escherichia coli* (VTEC) O157 has been carried out in Wales since 1990. The annual incidence has remained stable during the 9-year period (mean: 1.6 cases per 100,000 population); the rate is highest in children younger than 5 years of age. Blood in the stool is reported in fewer than half the cases, indicating the importance of screening all fecal specimens for VTEC O157.

Vero cytotoxin-producing *Escherichia coli* serogroup O157 (VTEC O157) was first recognized as a human pathogen in 1982 (1). Infection results in symptoms ranging from mild diarrhea to hemorrhagic colitis (abdominal pain, diarrhea, and blood in the stool). Hemolytic uremic syndrome (HUS), characterized by microangiopathic hemolytic anemia, thrombocytopenia, and renal failure, develops in 2% to 7% of cases (2). The number of laboratory isolations of VTEC O157 from human infections in England and Wales has risen from 76 in 1986 (3) to 1,087 in 1997 (4). However, the number of laboratories examining feces for VTEC O157 has increased in England, as have protocols emphasizing the importance of laboratory examination. Many surveillance networks worldwide have selection criteria for testing for VTEC O157, such as the presence of blood in the stools and clinical or age parameters (5). In contrast, population-based surveillance has been undertaken in Wales since February 1990, with all first-time acute-phase fecal specimens tested for VTEC O157 (6). The objectives of this surveillance are to measure the incidence of VTEC O157, identify outbreaks of infection, and describe the persons involved and the microbiologic characteristics of the isolates. We report on 9 years of surveillance through the end of 1998. Since the system is population-based, the figures differ from those in some published reports of specimens submitted to the reference laboratory (4,7,8).

Address for correspondence: Rachel Chalmers, PHLS CDSC (Wales), Abton House, Wedal Road, Cardiff CF4 3QX, United Kingdom; fax: 44-1-222-521-987; e-mail: rachel.chalmers @cdsc.wales.nhs.uk.

Fecal samples were cultured on Sorbitol MacConkey agar (Oxoid, Basingstoke, UK) and incubated at 37°C for 18 hours (3). Sorbitol nonfermenting colonies were tested for latex agglutination with O157 antiserum (Oxoid) and were biochemically confirmed as *Escherichia coli* by API 20E (BioMerieux sa69280 Marcy L'Etoile, France). Laboratories were asked to send all presumptive VTEC O157 isolates to the Laboratory of Enteric Pathogens, Central Public Health Laboratory, London, for confirmation, phage-typing, and Vero cytotoxin typing (8).

Cases, defined as "isolation of VTEC, confirmed by standard methods, from a fecal specimen submitted by a resident of Wales," were reported to the Public Health Laboratory Service Communicable Disease Surveillance Centre (CDSC) (Wales). Nine cases were excluded because the *E. coli* O157 isolate did not express Vero cytotoxin genes. Epidemiologic and clinical information was recorded on a standard structured questionnaire. Household contacts were screened where practicable and were included if they met the case definition. HUS, which in the United Kingdom is defined as renal impairment including oligouria and plasma creatinine elevated for age, microangiopathic hemolytic anemia, and thrombocytopenia, was diagnosed clinically.

The annual incidence was calculated by using as the denominator the mid-year population estimates for Wales (Office of National Statistics [ONS]), and the age and sex distribution of patients was calculated by using the mid-1996 population estimate (ONS). The Poisson distribution was used to calculate 95% confidence intervals (CI) for age-specific rates. To assess seasonality, the frequency of cases by month of onset was examined. (For asymptomatic cases, the date of the sample was used.) Incidence by health authority areas was calculated by using post-1996 boundaries. The proportions of cases with various symptoms were determined, and 95% CI were calculated by using standard error of proportions. The duration of illness (up to the date of interview), admission to hospital and length of stay, and proportion with HUS (95% CI) were calculated.

From 1990 through 1998, 415 cases were reported (mean = 1.6 per 100,000 population per year), with little change in incidence (1.0 per 100,000 population in 1994 to 2.8 per 100,000 population in 1995, when an outbreak of 49 cases occurred) (Table 1) (9). Seventy-four cases (17.8%) were part of six outbreaks involving Welsh residents (Table 1). Three of the outbreaks have been reported elsewhere (9-11). The remaining 341 (82.2%) were sporadic cases, of which 283 (83.0%) were index cases (the first reported from each household) (from 72.3% in 1998 to 96.2% in 1993). Fifty-eight (17.0%) sporadic cases were in household contacts; 26 of these patients had diarrhea, including five with blood in the stools.

Of 415 patients, 207 (49.9%) were males, ages 3 months to 89 years (mean = 25 years, median = 18 years, mode = 1). The incidence of VTEC O157 was highest in children younger than 5 years (8.8 per 100,000 population) (Table 2). The number of cases peaked in August, and more than half (227) of the cases occurred during July, August, and September. Only four cases occurred during December. Cases were reported from all five health authority areas in Wales. The highest incidence was in the northern and western areas (North Wales and Dyfed/Powys) (mean annual incidences 2.5 and 2.4 per 100,000 population, respectively). The lowest incidences were reported in the more densely populated areas of Gwent (1.1 per 100,000), Bro Taf (1.1 per 100,000), and Iechyd Morgannwg (1.0 per 100,000).

Of the 415 patients, 339 (81.7%, CI = 78.3%-85.7%) had diarrhea, 259 (62.4%, CI = 57.3%-66.7%) reported abdominal pain, and 192 (46.3%, CI = 41.3%-50.7%) had blood in the stool; 172 (41.4%, CI = 36.3%-45.7%) had hemorrhagic colitis. One third of the patients reported vomiting (32.3%, CI = 27.5%-36.5%) or feeling feverish (34.0%, CI = 29.5%-38.5%); 62 (14.9%, CI = 11.5%-18.5%) were asymptomatic. The highest proportion of asymptomatic cases was in the 25- to 34-year-old age group (18 [40.1%] of 44) who are often the caretakers of symptomatic patients;

Table 1. Occurrence and annual incidence of VTEC O157 in Wales, 1990–1998

| Year | Total no. of cases (rate per 100,000 population) | No. of sporadic cases (no. of index cases) | No. of outbreak cases | Outbreak summary (setting, mode of spread, phage and verotoxin types) |
|---|---|---|---|---|
| 1990 | 32 (1.1) | 28 (24) | 4 | Psychogeriatric ward, person to person, PT14, VT1&2. |
| 1991 | 39 (1.3) | 30 (28) | 9 | Day nursery, person to person, PT49, VT2. |
| 1992 | 41 (1.4) | 41 (33) | 0 | - |
| 1993 | 34 (1.2) | 26 (25) | 8 | Community, Meat from 1 shop, PT49, VT2. |
| 1994 | 29 (1.0) | 29 (24) | 0 | - |
| 1995 | 82 (2.8) | 33 (30) | 49 | Day nursery, person to person, PT2 VT2. |
| 1996 | 38 (1.3) | 38 (31) | 0 | - |
| 1997 | 55 (1.9) | 51 (41) | 3 | Home for the elderly mentally infirm, person to person, PT2, VT2. |
| | | | 1 | Part of a European outbreak |
| 1998 | 65 (2.2) | 65 (47) | 0 | |

Table 2. Age and sex distribution of cases of VTEC O157, Wales, 1990–1998

| Age range | Total | Male | Female |
|---|---|---|---|
| <1 | 24 (7.9, CI = 4.9-11.9) | 13 (8.3) | 11 (7.5) |
| 1-4 | 117 (9.0, CI = 7.4-10.7) | 71 (10.7) | 46 (7.2) |
| 5-14 | 56 (1.6, CI = 1.2-2.1) | 32 (1.8) | 24 (1.4) |
| 15-24 | 44 (1.4, CI = 1.0-1.8) | 19 (1.1) | 25 (1.6) |
| 25-34 | 44 (1.2, CI = 0.8-1.6) | 19 (1.0) | 25 (1.3) |
| 35-44 | 30 (0.9, CI = 0.6-1.2) | 14 (0.8) | 16 (0.9) |
| 45-54 | 33 (1.0, CI = 0.7-1.3) | 11 (0.6) | 22 (1.3) |
| 55-64 | 25 (0.9, CI = 0.6-1.5) | 10 (0.7) | 15 (1.1) |
| ≥65 | 35 (0.9, CI = 0.5-1.1) | 15 (0.9) | 20 (0.8) |
| Total (mean) | 415 (1.6, CI = 1.4-1.7) | 207 (1.6) | 208 (1.5) |

Figures in parentheses are mean annual rates per 100,000 population, followed by 95% confidence intervals (CI) for age-specific rates.

in 17 cases HUS developed (4.1%, CI = 2.4%-6.5%), age range: 1 to 50 years (mean = 9 years, median = 3 years); 10 HUS patients were less than 1 to 4 years of age, for a complication rate in this age group of 8.5%.

Diarrheal illness lasted as long as 330 days (median and mode = 6 days); 118 (28.4%) patients were admitted to hospital. The length of stay, first recorded in 1994, was from 1 to 71 days (mode 1 day, median 4.0 days). The highest rate of hospitalization was among those >65 years old (25 [62.5%] of 40). The mean annual proportion of index cases hospitalized was 36.1% (24.0% in 1993 to 48.5% in 1992). From 1994 through 1998, only one person, an 88-year-old woman with diarrhea, died as a result of the infection.

Three hundred seventy-eight (91.1%) isolates were sent to the Laboratory of Enteric Pathogens for confirmation and typing. Of these, 62 (16.4%, CI = 6.4%-26.4%) had both verotoxin type (VT) 1 and VT2 genes, and 316 (83.6%, CI = 79.9%-87.3%) had VT2 only. Isolates belonged to at least 19 phage types (PT). The two most common PT were PT2 (160 isolates [42.3%]) and PT49 (48 isolates [12.7%]). Other PT accounting for 5% (19) or more isolates were PT1, PT4, PT8, and PT14. PT2 was the most common type in each year, with the exception of 1993, when PT49 predominated. PT and verotoxin type were linked: VT2-only strains included 98% (158 of 160) of the PT2 and all the PT49 isolates.

No relationship was found between the major PTs and clinical symptoms. More cases with strains producing VT1+2 had hemorrhagic colitis (39 [63.9%] of 61) than cases with VT2 only (120 [42.9%] of 280) (relative risk = 1.69, 95% CI = 1.18-1.88). In contrast, 16 of the 17 isolates from cases of HUS had the VT2 gene only. These isolates were predominantly PT2 (n = 10), but also included PT49 (n = 4), PT21 (n = 1) and RDNC (n = 1).

Foreign travel in the week before onset of symptoms was reported by 37 (8.9%) patients (0 in 1990 to 12 in 1998 [18.5%] of cases). The PTs among those who had traveled abroad differed from the overall pattern, the most common being PT8 (10 cases), RDNC (5 cases), and PT21 (4 cases).

Population-based surveillance of VTEC O157 in Wales has been undertaken since 1990 and is the most complete in the world. There is no evidence that pathology referrals have changed during the study period. General practitioners (primary-care physicians) were given no specific incentives for submitting specimens. Palmer et al. (12) showed that in 1996, 26% patients with suspected food poisoning attending general-practitioner clinics submitted fecal specimens. This is similar to the 27% reported during a study of patients with infectious gastroenteritis reporting to general practitioners in England (13). Although VTEC O157 is regarded as an emerging pathogen, in Wales its incidence has remained stable through 1998, and VTEC O157 is a rare (1.6 cases per 100,000 population) but serious disease.

Public health policy concerning VTEC O157 has been driven by the circumstances surrounding outbreaks (14). However, in Wales most cases (82.2%) occur sporadically, and because all first-time specimens and PTs are examined and epidemiologic investigations are conducted, it is unlikely that outbreaks were missed. The surveillance data, as well as providing a background against which to measure changes in incidence, have provided useful information about VTEC O157 infections. The presence of blood in the stool is often used in many countries as a criterion for examining for VTEC O157, yet fewer than half the Welsh patients reported the presence of blood, demonstrating the value of screening all acute-phase fecal specimens. Although 14.9% of cases were asymptomatic, the risk for transmission is still present because of the low infectious dose (11).

Strains of VTEC O157 can be differentiated rapidly by PT and Vero cytotoxin typing, although even from apparently sporadic cases a large number of isolates belonged to a few types, predominantly PT2/VT2 and PT49/VT2. Determining the VT produced appears to be a microbiologic marker of severity, since hemorrhagic colitis was more often associated with VT1+2 strains, and all the cases complicated by HUS had VT2-only strains. These strains are consistently more prevalent than other VT types in cases of HUS in the United Kingdom (7) and elsewhere. Demonstration of VT by phenotypic tests or the presence of VT genes is definitive for VTEC O157. The presence of the H7 antigen is closely associated with VT positivity but is of secondary importance, as a significant minority of VTEC O157 isolated in England and Wales (14%-20% in 1992-1994) are nonmotile (8). There was some annual variation in PTs, although as in England the predominant phage type was PT2.

As with other surveillance reports, the highest incidence was in children younger than 5 years of age (8). Although fecal specimens are more likely to be available for this age group, the isolation rate is also high (6), and person-to-person spread is most likely (15). In Wales, person-to-person spread was the most important factor in four out of five outbreaks, including those in the children's day nurseries, which were the setting for the two largest outbreaks.

Continued surveillance for VTEC O157 will provide timely reporting of cases and detection and containment of outbreaks. Ongoing surveillance over the last 9 years in Wales has provided valuable information about VTEC O157 infections and demonstrated the wide range of associated clinical illness.

### Acknowledgments

The authors thank the microbiology laboratories and their link personnel, District Environmental Health Departments, Health Authority Consultants in Communicable Disease Control, and the Welsh Office Standing Specialist Advisory Group (Microbiology). Unipath/Oxoid organized the distribution of reagents to participating laboratories.

This work was funded by grants from the Department of Health, London (DH code 145 and 254) and a PHLS Small Projects grant.

Dr. Chalmers is a senior scientist with the U.K. Public Health Laboratory Service Communicable Disease Surveillance Centre in Cardiff. She has a research background in veterinary microbiology and protozoology and current interests in the epidemiology of occupational and food- and waterborne zoonoses.

### References

1. Riley LW, Remis RS, Helgerson SD, McGee HB, Walls J, Davis BR, et al. Hemorrhagic colitis associated with a rare *Escherichia coli* serotype. N Engl J Med 1983;308:681-5.
2. Tarr PI. *Escherichia coli* O157:H7: clinical, diagnostic and epidemiological aspects of human infection. Clin Infect Dis 1995;20:1-10.
3. Advisory Committee on the Microbiological Safety of Food. Report on Vero cytotoxin-producing *Escherichia coli.* London: The Committee; 1995.
4. Vero cytotoxin producing *Escherichia coli* O157 in England and Wales. Commun Dis Rep CDR Wkly 1998;8:169.
5. Parry SM. The incidence and sources of Vero cytotoxin producing *Escherichia coli* O157 in Wales and the English Borders [Ph.D. thesis]. University of Leeds; 1997.
6. Salmon RL, Smith RMM. How common is *Escherichia coli* O157 and where is it coming from? Total population surveillance in Wales 1990-1993. In: Karmali MA, Golglio AG, editors. Recent advances in Vero-cytotoxin-producing *Escherichia coli* infections. Amsterdam: Elsevier Science BV; 1994.
7. Thomas A, Chart H, Cheasty T, Smith HR, Frost JA, Rowe B. Vero cytotoxin-producing *Escherichia coli*, particularly serogroup O157, associated with human infections in the United Kingdom: 1989-1991. Epidemiol Infect 1993:110;591-600.
8. Thomas A, Cheasty T, Frost JA, Chart H, Smith HR, Rowe B. Vero cytotoxin-producing *Escherichia coli*, particularly serogroup O157, associated with human infections in England and Wales: 1992-1994. Epidemiol Infect 1996:117;1-10.
9. Al-Jader L, Salmon RL, Walker AM, Williams HM, Willshaw GA, Cheasty T. An outbreak of Vero cytotoxin producing *Escherichia coli* O157 in a private nursery in North Wales: lessons for prevention. Arch Dis Child 1999: 81;60-3.
10. Furtado C, Rojas A, Pebody R, Nylen G, McCarthy N, Donnelly M, et al. Investigation of an outbreak of *Escherichia coli* O157 in Europe 1997. Journal d'Epidemiologie de Terrain le Bulletin d'Epiter;11;90.
11. Willshaw GA, Thirwell J, Jones AP, Parry S, Salmon RL, Hickey M. Vero cytotoxin-producing *Escherichia coli* O157 in beefburgers linked to an outbreak of diarrhoea, haemorrhagic colitis and haemolytic uraemic syndrome in Britain. Letts Appl Microbiol 1994;19:304-7.
12. Palmer S, Houston H, Lervy B, Ribiero D, Thomas P. Problems in the diagnosis of foodborne infection in general practice. Epidemiol Infect 1996;117:479-84.
13. Wheeler JG, Sethi D, Cowden JM, Wall P, Rodrigues LC, Tompkins DS, et al. Study of infectious intestinal disease in England: rates in the community, presenting to general practice, and reported to national surveillance. BMJ 1999;318:1046-50.
14. Pennington Group. Report on the circumstances leading to the 1996 outbreak of infections with *E. coli* O157 in central Scotland. The implications for food safety and the lessons to be learned. Edinburgh: The Stationery Office; 1997.
15. Parry SM, Salmon RL. Sporadic STEC infection: secondary household transmission in Wales. Emerg Infect Dis 1998;4:657-61.

## References

[PDF_00361] Al-Jader L., Salmon R. L., Walker A. M., Williams H. M., Willshaw G. A., Cheasty T. (1999). Outbreak of Escherichia coli O157 in a nursery: lessons for prevention.. Arch Dis Child.

[PDF_00375] Palmer S., Houston H., Lervy B., Ribeiro D., Thomas P. (1996). Problems in the diagnosis of foodborne infection in general practice.. Epidemiol Infect.

[PDF_00330] Riley L. W., Remis R. S., Helgerson S. D., McGee H. B., Wells J. G., Davis B. R., Hebert R. J., Olcott E. S., Johnson L. M., Hargrett N. T. (1983). Hemorrhagic colitis associated with a rare Escherichia coli serotype.. N Engl J Med.

[PDF_00334] Tarr P. I. (1995). Escherichia coli O157:H7: clinical, diagnostic, and epidemiological aspects of human infection.. Clin Infect Dis.

[PDF_00351] Thomas A., Chart H., Cheasty T., Smith H. R., Frost J. A., Rowe B. (1993). Vero cytotoxin-producing Escherichia coli, particularly serogroup O 157, associated with human infections in the United Kingdom: 1989-91.. Epidemiol Infect.

[PDF_00356] Thomas A., Cheasty T., Frost J. A., Chart H., Smith H. R., Rowe B. (1996). Vero cytotoxin-producing Escherichia coli, particularly serogroup O157, associated with human infections in England and Wales: 1992-4.. Epidemiol Infect.

[PDF_00378] Wheeler J. G., Sethi D., Cowden J. M., Wall P. G., Rodrigues L. C., Tompkins D. S., Hudson M. J., Roderick P. J. (1999). Study of infectious intestinal disease in England: rates in the community, presenting to general practice, and reported to national surveillance. The Infectious Intestinal Disease Study Executive.. BMJ.

